# *K* Important Neighbors: A Novel Approach to Binary Classification in High Dimensional Data

**DOI:** 10.1155/2017/7560807

**Published:** 2017-12-11

**Authors:** Hadi Raeisi Shahraki, Saeedeh Pourahmad, Najaf Zare

**Affiliations:** ^1^Department of Biostatistics, School of Medicine, Shiraz University of Medical Sciences, Shiraz, Iran; ^2^Bioinformatics and Computational Biology Research Center, Shiraz University of Medical Sciences, Shiraz, Iran; ^3^Infertility Research Center, Shiraz University of Medical Sciences, Shiraz, Iran

## Abstract

*K* nearest neighbors (KNN) are known as one of the simplest nonparametric classifiers but in high dimensional setting accuracy of KNN are affected by nuisance features. In this study, we proposed the *K* important neighbors (KIN) as a novel approach for binary classification in high dimensional problems. To avoid the curse of dimensionality, we implemented smoothly clipped absolute deviation (SCAD) logistic regression at the initial stage and considered the importance of each feature in construction of dissimilarity measure with imposing features contribution as a function of SCAD coefficients on Euclidean distance. The nature of this hybrid dissimilarity measure, which combines information of both features and distances, enjoys all good properties of SCAD penalized regression and KNN simultaneously. In comparison to KNN, simulation studies showed that KIN has a good performance in terms of both accuracy and dimension reduction. The proposed approach was found to be capable of eliminating nearly all of the noninformative features because of utilizing oracle property of SCAD penalized regression in the construction of dissimilarity measure. In very sparse settings, KIN also outperforms support vector machine (SVM) and random forest (RF) as the best classifiers.

## 1. Introduction

The aim of classification methods is to assign true label to a new observation. Despite the fact that classification is one of the oldest statistical methods, finding the mechanism by which new observations are classified with the lowest error is still challenging. Although Fernández-Delgado et al. showed that there was no classifier which has the highest accuracy in all the situations, they present random forest (RF) and support vector machine (SVM) as the best classifiers among 182 classifiers [1].

*K* nearest neighbors (KNN) are known as one of the simplest nonparametric classifiers. For a fixed value of *k*, KNN assigns a new observation to the class of majority of the *k* nearest neighbors [2, 3]. Nevertheless, in high dimensional setting, it is affected by nuisance (noninformative) features and suffers from “*curse of dimensionality*” [4–6]. In recent years, the effect of the curse of dimensionality on KNN has been studied by many authors. For example, Pal et al. showed that, in high dimensional setting, KNN classifier misclassifies about half of the observations [3, 7] and Lu et al. have noted that the nature of sparsity in high dimensional situation can lead to unstable results [5]. As a result of dimensionality curse, it has been argued by some authors that nearest neighbor can become ill defined because all pairwise distances concentrate around a single value (distance concentration) [3, 4, 7]. Beyer et al. stated that distance concentration can occur even with as few as 15 dimensions [7]. In 2010, Radovanović et al. introduced* k-occurrences* as follows: “the number of times a point appears among the *k* nearest neighbors of other points in a data set.” They also showed the deleterious impact of points with very high *k*-occurrences called* hubs* [6]. Another challenge in KNN method is about ties when sample size is small. Empirical practice showed that *k* is not greater than square root of number of training data items [2]. Therefore for binary classification, when *k* is even, there is the chance of ending with a tie vote. To eliminate this challenge, KNN only considers odd numbers [2, 8].

In the last decade, dimension reduction techniques as a remedial method for classification with KNN in high dimensional settings have been more attentive. Fern and Brodley proposed random projection, which was based on a random matrix. This random matrix projects the data along a subspace with lower dimension, so KNN classifier utilizes the reduced subspace for classification task [9]. Deegalla and Boström proposed principal component based projection when the number of PCs was lower than data dimensions. They recommended using aforementioned PCs instead of initial features for dissimilarity measure construction and finding the *k* nearest neighbors [10]. Another popular approach is to employ a threshold (so called hard threshold) and truncate less important features. In this approach, only features greater than the threshold are contributed to KNN classifier [11]. Pal et al. proposed a new dissimilarity measure based on mean absolute difference of distances (MADD) to cope with curse of dimensionality [3]. Finally in 2013, Lu et al. stated that, in the sparse situations to enhance accuracy, a classifier should combine both linearity and locality information [5].

In this manuscript, we suggest a hybrid method called* K important neighbors* (KIN) that implements* smoothly clipped absolute deviation* (SCAD) regression and uses a function of the obtained coefficients as weights in construction of dissimilarity measure. Proposed method combines information of features employing logit link function (i.e., linearity information) and distances (i.e., locality information) in the dissimilarity measure, thereby leading to both feature selection and classification. In facing ties, KIN assign new observation to a class with lower amount of dissimilarity measure.

The rest of this paper is organized as follows: Section 2 presents a brief description about KNN, SCAD penalized regression, random forest (RF), and support vector machine (SVM). In Section 3, we present our proposed method.  Section 4 compares the accuracy of KIN with KNN, RF, and SVM using simulation studies and benchmark data sets and finally, we provide discussion about the proposed classifier and conclude this manuscript in Section 5.

## 2. **Statistical Methods**

### 2.1. *K* Nearest Neighbors (KNN)

The *k* nearest neighbors classifier assigns a new observation into a class with majority votes in *k* nearest neighbors [12, 13]. The dissimilarity measure in KNN is usually defined in terms of Minkowski distance as follows:

(1) $$\begin{array}{c} d\left(\;x_{a},x_{b}\;\right)={\left(\;\sum_{j=1}^{p}{\left(\;x_{aj}-x_{bj}\;\right)}^{q}\;\right)}^{1/q}, \end{array}$$

where *p* is the number of features, *q* is a positive constant (usually 1 or 2), and *d*(*x*_*a*_, *x*_*b*_) is distance between *a* and *b* points. Optimum amounts of *k* (number of neighbors) can be obtained using cross validation technique [2, 8].

### 2.2. Smoothly Clipped Absolute Deviation (SCAD)

Variable selection is one of the key tasks in high dimensional statistical modeling. Penalized likelihood approach by handling curse of dimensionality performs estimation and variable selection simultaneously [14]. Smoothly clipped absolute deviation (SCAD) logistic regression proposed for feature selection in high dimension and low sample size settings by Fan and Li is as follows:

(2) Lβ;λ=lnβ+λ∑j=1ppβj,pβj=Iβj≤λ+3.7λ−βj+2.7λIβj>λ,

where **β**^*T*^ = (*β*_1_, *β*_2_, …, *β*_*P*_) is vector of coefficients, *l*_*n*_(*β*) is maximum likelihood estimator of regression model, *p*(*β*) is penalty function, and *λ* is a positive constant called regularization (tuning) parameter [15, 16]. The amount of penalty depends on *λ* which is estimated using 5- or 10-fold cross validation technique. SCAD has good properties of both best subset and ridge regression which yield continuous and unbiased solutions. Moreover, it can estimate nuisance features as zero and signal (informative) features as nonzero with probability very close to one. This advantage of SCAD regression called “oracle” property and means that SCAD is able to estimate coefficients of all the features truly with probability which tends to one [15]. In short, SCAD selects the correct model as well as we hope, even in very sparse and low sample size situations.

### 2.3. Random Forest (RF)

Random forest (RF) is a method for regression or classification that is based on an ensemble of unpruned trees. In RF, each tree is built on a bootstrap sample (almost two-thirds of the observation) and grows via a random sample of features at each split. For classification tasks, this random sample is the square root of the total features. This is repeated hundreds of times for building a forest. Optimum number of trees in RF can be estimated by out of bag error and the class with majority votes is considered as the class of new observation [8, 17]. In the current study, randomForest package was used and default number of trees set at 500.

### 2.4. Support Vector Machine (SVM)

The aim of support vector machine (SVM) is to find a line which maximizes the margin between two classes. To attain this goal, SVM incorporates kernel trick that allows the expansion of the feature space. Also, support vector refers to any observation which for its class lies on the wrong side of the margin. Expansion of the feature space depends on the number of support vectors estimated by cross validation [8, 18]. In the current study, we used linear kernel and cost function ranging between 0.001 and 5 in e1071 package.

## 3. *K* Important Neighbors (KIN) Algorithm for Binary Classification

Suppose that *R* = {(*y*_*i*_, *x*_*i*_), *i* = 1, …, *n*_*r*_} is a training data set and *y*_*i*_ ∈ {0, 1} denotes class membership and vector of **p** predictor features for *i*th observation represented as **x**_**i**_ = (*x*_*i*1_, *x*_*i*2_, …, *x*_*ip*_).

After random division of data into training and testing set, SCAD logistic regression was fitted on training data set which leads to estimating coefficients of nuisance features to be exactly zero. In the next step, the contribution (importance) of each feature is calculated using the following formula:

(3) $$\begin{array}{c} w_{j}=\frac{\left|\beta_{j}\right|}{\sum_{j=1}^{p}\left|\beta_{j}\right|}\;j=1,2,\ldots ,p, \end{array}$$

where *β*_*j*_ is coefficient of *j*th feature in SCAD logistic regression. By imposing the obtained vector of contributions into Euclidian distance, we introduce our proposed dissimilarity measure as follows:

(4) $$\begin{array}{c} d\left(\;x_{a},x_{b}\;\right)={\left(\;\sum_{j=1}^{p}{w_{j}\left(x_{aj}-x_{bj}\right)}^{2}\;\right)}^{1/2}, \end{array}$$

where *d*(*x*_*a*_, *x*_*b*_) is distance between *a* and *b* points.

In the next stage, we obtain optimum number of neighbors (*k*) using the proposed dissimilarity measure and considering both even and odd values. A new observation was assigned to class one (*y* = 0) if *k*_1_ > *k*_2_ and assigned to class two (*y* = 1) if *k*_1_ < *k*_2_ where *k*_*i*_ is number of observations in the *i*th class among *k* nearest neighbors. When a tie occurs (*k*_1_ = *k*_2_) assignment rule is as follows:

(5) $$\begin{array}{c} y=\left\{\begin{array}{cc} 0, & if\;\;\underset{k_{1}}{\sum }{\left(\sum_{j=1}^{p}{w_{j}\left(x_{aj}-x_{bj}\right)}^{2}\right)}^{1/2}<\underset{k_{2}}{\sum }{\left(\sum_{j=1}^{p}{w_{j}\left(x_{aj}-x_{bj}\right)}^{2}\right)}^{1/2},\\ 1, & if\;\;\underset{k_{1}}{\sum }{\left(\sum_{j=1}^{p}{w_{j}\left(x_{aj}-x_{bj}\right)}^{2}\right)}^{1/2}>\underset{k_{2}}{\sum }{\left(\sum_{j=1}^{p}{w_{j}\left(x_{aj}-x_{bj}\right)}^{2}\right)}^{1/2}; \end{array}\right. \end{array}$$

it means assigning new observation into class with lower dissimilarity index.

**Figure 1:**
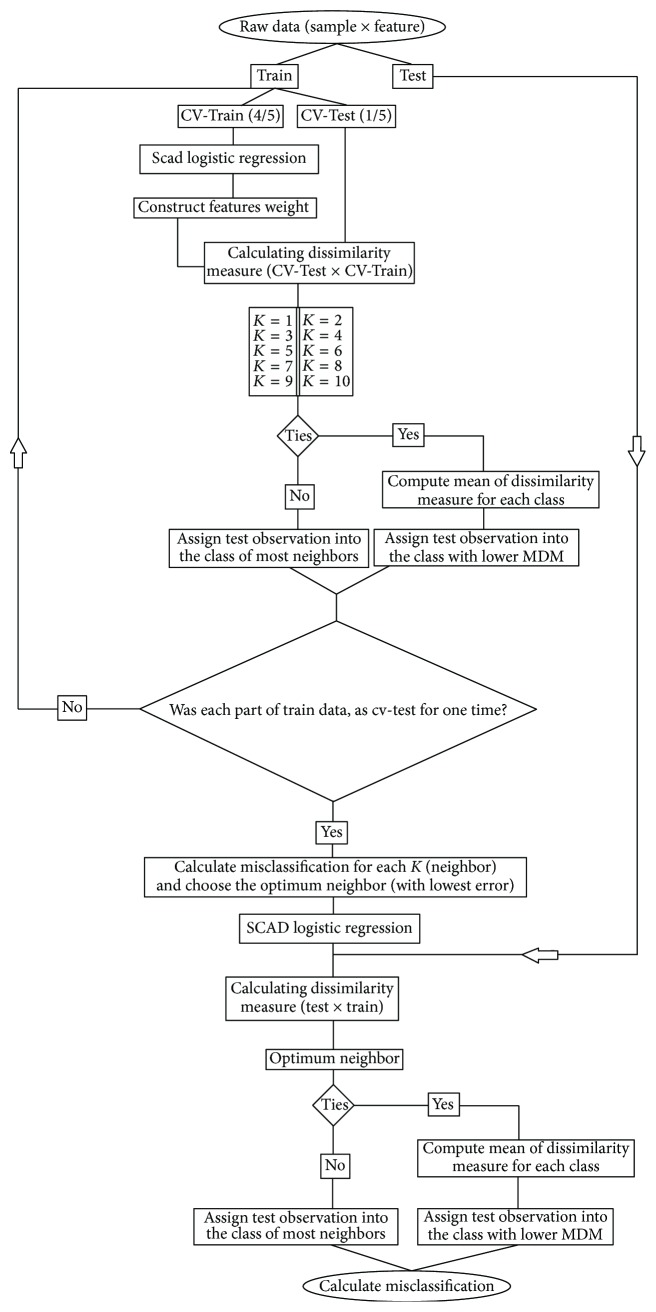
*K* important neighbors (KIN) algorithm for classification.

To avoid a significant decrease in sample size of each fold, 5-fold cross validation was implemented for choosing optimum number of neighbors (*k*) because sample size in training data set may be as small as 30. In 5-fold cross validation technique, training data set (40% of total sample size in the current study) may randomly be divided into 5 equal parts. Each time one part is considered as validation while another part was used for training the model. This is repeated 5 times, so all the parts are used just once as validation set and mean error of the 5 repeat was calculated as cross validation error. Finally, after obtaining the optimum value *k* of neighbors and using a matrix of dissimilarity measure, testing set (60% of total sample size in the current study) was assigned into the groups. In order to calculate misclassification rate (MC), the following formula was used:

(6) $$\begin{array}{c} MC=\overset{1}{\underset{y=0}{\sum }}\pi_{y}\frac{m_{y}}{n_{y}}, \end{array}$$

where *π*_*y*_, *m*_*y*_, and *n*_*y*_ represent ratio of observation, number of misclassifications, and sample size of the desired class, respectively. The algorithm used is described in a flowchart and displayed in Figure 1.

## 4. Numerical Comparisons

### 4.1. Simulation Framework

In the following scenarios, the misclassification rate of the proposed method called KIN was numerically compared with the traditional KNN, random forest (RF), and support vector machine (SVM) methods. The reason for the choice of RF and SVM is that they are the best among all of the current classifiers. All the simulations are performed in R 3.1.3 software and 5-fold cross validation was used to estimate optimum number of trees and support vectors in RF and SVM, respectively, or optimum number of neighbors in KNN and KIN methods.

We simulated 250 data sets for each scenario, comprising 100 or 200 observations from the model *Y* ~ Bernoulli(*p*(*X*^*T*^*β*)), where *Y* denotes class membership, *p*(*u*) = (exp⁡(*u*))/(1 + exp⁡(*u*)), and *X* is a vector of features and each feature has standard normal distribution. Let *β* = (*β*_non zero_, *β*_zero_), where *β*_non zero_ is a vector of 1 for their odd components and 2 for their even components and *β*_zero_ is vector of zero components. Degree of sparsity was determined by *β*_zero_/*β* which was considered as 90, 95, or 98% and number of features was set to 100, 300, or 500. Moreover to assess effect of correlation between features on the accuracy of the proposed classifier, a kind of autoregressive correlation was used. In this correlation pattern, the closer two variables are together, the more correlation is between them as follows: the correlation between *x*_*j*′_ and *x*_*j*_ (two arbitrary features) was considered as *ρ*^|*j*′ − *j*|^ where *ρ* was 0.8 or 0.4. In all the scenarios, we split simulated data set randomly into training and testing set with ratio of 40% and 60%, respectively. The reason for choosing smaller sample size for training set was assessing the accuracy of the proposed model compared to the best classifiers in low sample size settings.

### 4.2. Simulation Results

Table 1 compares the average misclassification rate of KNN and KIN in all the scenarios. The results indicate that using proposed KIN improves classification accuracy of KNN on average of 4.9, 5.9, and 8.8% when degree of sparsity is 90, 95, and 98%, respectively. In Table 1, we also demonstrate oracle property of KIN in the number of false positive variables (#FP) columns. The mean number of false positive variables was 1.9, 2.7, and 3.0 when numbers of variables were 100, 300, and 500, respectively. In fact, the proposed method successfully eliminates 98.8, 98.7, and 98.8% of noisy features in 90, 95, and 98% degree of sparsity scenarios, respectively. Our results indicated that KIN also has good performance in terms of assigning true weight to signal (nonzero) features. We called this* true contribution* (TC). Table 1 showed that the average true contributions were 80.2, 77.1, and 69.8% for 100, 300, and 500 predictors, respectively.

**Figure 2:**
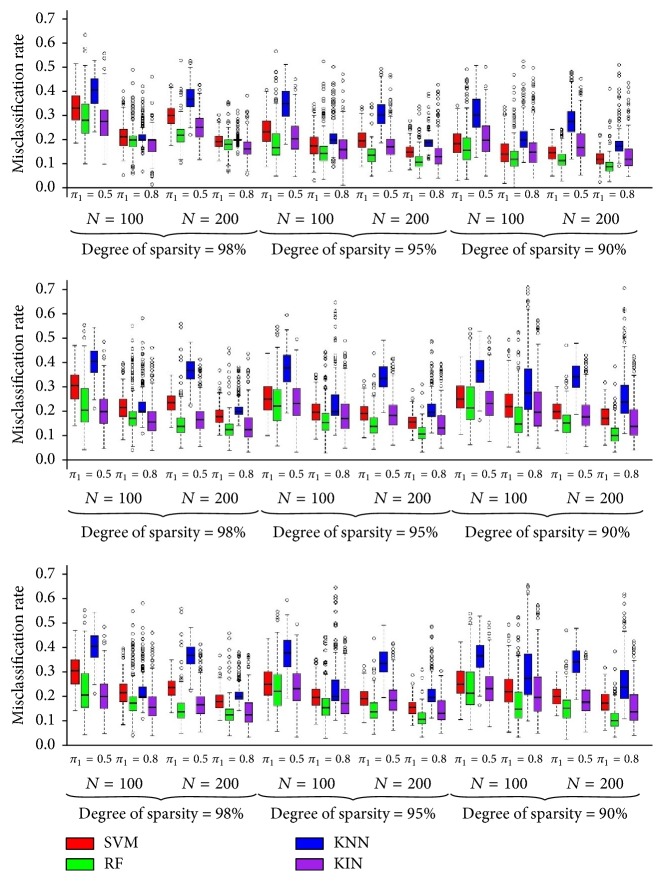
Misclassification rate of proposed KIN versus SVM, RF, and KNN for 100, 300, and 500 (up to down) features (*N* indicates sample size).

Misclassification (MC) rate of KIN was compared to KNN, RF, and SVM for the above scenarios in Figure 2. This figure indicates that the superiority of proposed KIN rather than KNN is obvious in all the situations. Also in very sparse situations where degree of sparsity is 98%, KIN outperforms RF and SVM most of the times and has comparable accuracy in the other sparse situations. We also introduced the probability of achieving the maximum accuracy (PAMA) for each of the classifiers, as the number of scenarios for which the classifier achieves the highest accuracy (among 4 classifiers) is divided by the total number of scenarios.  Table 2 shows the values of PAMA for each classifier in different degrees of sparsity. We can infer that the probability of achieving the maximum accuracy in KIN increases when degree of sparsity increases to 100% as the highest amount of PAMA for KIN is 66.7%, where only 2% of features are signal. Note that PAMA values are very far from 100% indicating that no classifier is the best for all settings.

Another useful measure which can be taken into consideration with very near accuracy from the best classifier is the probability of achieving more than 95% of the maximum accuracy (P95). The P95 for each classifier is estimated as the number of scenarios in which it achieves 95% or more of the maximum accuracy (among 4 classifiers), divided by the total number of scenarios. Once again, we can see that the proposed KIN is the best classifier in terms of P95 for very sparse situations and totally, KIN is dominant over the SVM and KNN (Table 2).

### 4.3. Benchmark Data Sets

In order to further assess the KIN classifier, we analyzed five data sets. The first two data sets were taken from the UCI machine learning repository (http://archive.ics.uci.edu/ml/datasets.html). Prostate cancer data set was from SIS package (only the first 600 features) and colon cancer data set from HiDimDA package in R software [19, 20]. We also used liver transplant data set as described in [21] to examine the accuracy of KIN in very unbalance class membership situations. In liver transplant data set, only 11% of patients were dead (*y* = 1) and the rest were alive (*y* = 0). In these data sets, instead of using specific training and testing set, we used random partitioning of the whole data and for each of them, we form 200 training and testing sets and average accuracy rate was computed over these 200 partitions.

The results of classification on benchmark data sets are summarized in Table 3. For data sets with small or moderate number of features such as liver transplant, connectionist bench, and ozone, there was ignorable difference between accuracy of KIN and that of KNN. The accuracy of KIN was higher than KNN in very high dimensional data sets (prostate and colon cancer). Although simulation results showed that accuracy of KIN is affected by data sets' degree of sparsity, in comparison to SVM and RF as the best classifiers, proposed KIN has comparable accuracy in high dimension and low sample size (in training data set) settings.

## 5. Discussion

Regarding the idea of Lu et al. that demonstrates how to enhance accuracy, a classifier should combine both linearity and locality information [5], we proposed a novel dissimilarity measure for *K* nearest neighbors classifier. To avoid deleterious effects of curse of dimensionality on KNN method, all the proposed solutions up to now can be summarized into two main categories: (1) dimension reduction which is based on feature selection or feature extraction [22–25] or (2) introducing a new dissimilarity measure [3]. From this perspective, assigning KIN in both of the above categories can be justified. By handling curse of dimensionality, KIN is capable classifier to overcome distance concentration and does not allow creating hubs. Moreover, managing ties challenge in small sample size leads to stable results.

Proposed feature extraction techniques for dimension reduction in KNN such as principal component analysis [10], linear discriminant analysis [26], locality preserving projections [27], random projection [9, 10], and nearest feature subspace [24] have two main defects: (1) feature extraction does not explain 100% of features information, thereby leading to waste of some valuable information and (2) since extracted features are combination of both signals and noises, the importance of each feature in classification may not be clearly achievable.

Our idea in present study is very close to Chan and Hall approach in 2009. They suggested truncated nearest neighbor which implements feature selection via a threshold before classification task [11]. Fan and Li called this threshold hard threshold and proposed a threshold in SCAD regression as SCAD threshold [15]. Hence, against truncated nearest neighbor, KIN use SCAD threshold that simultaneously satisfies unbiasedness and sparsity [15]. Another important difference between two aforementioned methods is that selected features in KIN do not have the same contribution in construction of dissimilarity measure which comprise an obvious advantage. Although MADD index as a novel dissimilarity measure for KNN classifier has good accuracy in high dimensional problems, compared to our hybrid dissimilarity measure, it does not take into consideration importance of features and is only based on distances [3]. Considering this shortcoming, we can infer that, as the degree of sparsity tends to one, MADD index becomes weaker but KIN becomes stronger in terms of accuracy.

Consequently, imposing features contribution as a function of SCAD coefficients on Euclidean distance (novelty of the present study) leads to four good properties:

1. It uses information of both variables and locations instead of usual dissimilarity measure in KNN which ignores information of features.
2. It performs dimension reduction because only those variables that contribute in construction of dissimilarity measure have nonzero coefficients.
3. It increases accuracy by eliminating noisy features from classification procedure and considering relative importance of the signal features.
4. It does not choose necessarily the *k* nearest neighbors. The nature of this hybrid measure leads to choosing *k* important neighbors (KIN); that helps to find more complex patterns in the presence of a huge number of noisy features.

### 5.1. Conclusion

In summary, KIN has a good performance in terms of both accuracy and dimension reduction. The proposed KIN also in very sparse settings outperforms support vector machine (SVM) and random forest (RF) as the best classifiers. The KIN approach was found to be capable of eliminating nearly all of the noninformative features because of utilizing oracle property of SCAD penalized regression in the construction of dissimilarity measure. What distinguishes KIN from KNN, SVM, and RF classifiers is that not only does the proposed KIN perform classification task, but it can also perform feature selection. In fact, KIN implements classification only with very small subgroup of features which can affect class assignment.

## Figures and Tables

**Table 1:** Misclassification rate (MC) of KIN versus KNN in simulation study.

Number of features	Degree of sparsity (%)	Sample size	Ratio of the first class	*R* = 0.4^|*j*−*j*′|^				*R* = 0.8^|*j*−*j*′|^			
				KNN	KIN			KNN	KIN		
				MC	MC	TC (%)	#FP	MC	MC	TC (%)	#FP
100	98%	*N* = 100	0.5	43.2	30.7	66%	2.7	40.0	27.5	69%	2.5
			0.8	21.4	19.1	71%	1.4	21.6	17.9	65%	1.5
		*N* = 200	0.5	40.2	27.6	82%	2.5	37.2	25.4	85%	2.1
			0.8	20.1	17.8	79%	1.3	20.2	16.6	83%	1.1
	95%	*N* = 100	0.5	39.6	28.0	76%	2.4	35.0	21.0	84%	2.1
			0.8	21.1	19.1	64%	1.7	21.0	16.3	83%	1.7
		*N* = 200	0.5	36.8	23.8	87%	3.0	30.8	17.9	88%	2.6
			0.8	20.0	16.5	87%	1.9	19.2	14.2	92%	1.3
	90%	*N* = 100	0.5	39.1	33.9	68%	2.1	30.8	20.5	89%	1.5
			0.8	21.6	22.0	59%	1.6	20.8	16.5	89%	1.5
		*N* = 200	0.5	36.9	28.0	87%	2.7	28.1	18.5	92%	2.1
			0.8	20.0	18.6	85%	1.9	18.4	14.3	94%	1.6

300	98%	*N* = 100	0.5	43.5	35.4	68%	2.8	40.1	20.4	82%	2.6
			0.8	22.1	21.7	60%	2.2	22.9	17.0	80%	2.4
		*N* = 200	0.5	41.2	25.3	77%	5.3	36.6	17.4	87%	3.2
			0.8	20.8	18.3	77%	3.1	21.0	14.9	88%	2.7
	95%	*N* = 100	0.5	43.6	41.9	57%	2.1	37.6	23.8	85%	2.1
			0.8	22.3	23.3	54%	2.4	23.5	19.5	83%	2.1
		*N* = 200	0.5	41.8	33.0	79%	3.6	33.8	19.4	90%	2.8
			0.8	21.1	21.9	70%	3.0	21.2	16.7	90%	2.6
	90%	*N* = 100	0.5	43.6	41.8	53%	2.3	36.6	23.7	85%	2.1
			0.8	32.1	29.7	64%	2.1	30.3	22.9	85%	1.9
		*N* = 200	0.5	41.5	33.1	76%	3.7	34.1	19.9	91%	2.7
			0.8	28.3	28.0	77%	3.5	26.2	16.9	92%	2.5

500	98%	*N* = 100	0.5	44.7	41.3	58%	2.5	39.9	21.6	82%	2.5
			0.8	22.5	22.5	51%	2.3	23.1	17.1	78%	2.5
		*N* = 200	0.5	43.5	29.0	75%	4.9	37.9	17.2	87%	4.0
			0.8	20.5	18.9	69%	3.3	21.3	14.8	88%	3.2
	95%	*N* = 100	0.5	45.7	44.8	44%	6.2	38.8	29.3	74%	2.3
			0.8	23.2	25.3	40%	2.3	26.6	22.5	74%	2.2
		*N* = 200	0.5	44.5	40.5	69%	3.3	35.8	22.3	88%	3.3
			0.8	21.1	24.6	66%	3.1	21.9	18.1	88%	3.0
	90%	*N* = 100	0.5	45.7	45.1	46%	2.5	39.3	26.0	83%	2.0
			0.8	28.2	32.2	50%	2.3	29.7	25.4	77%	2.1
		*N* = 200	0.5	46.5	45.2	58%	2.8	36.9	29.5	86%	3.3
			0.8	26.1	30.3	59%	3.2	21.8	23.2	86%	2.8

**Table 2:** Comparison of methods in terms of probability of achieving the maximum accuracy (PAMA) and probability of achieving more than 95% of the maximum accuracy (P95).

Degree of sparsity	Method	PAMA	P95
90%	Random forest (RF)	45.8%	66.7%
	Support vector machine (SVM)	50.0%	87.5%
	*K* nearest neighbors (KNN)	0.0%	20.8%
	*K* important neighbors (KIN)	8.3%	41.7%

95%	Random forest (RF)	50%	79.2%
	Support vector machine (SVM)	29.2%	79.2%
	*K* nearest neighbors (KNN)	0.0%	29.2%
	*K* important neighbors (KIN)	20.8%	75%

98%	Random forest (RF)	20.8%	75%
	Support vector machine (SVM)	12.5%	41.7%
	*K* nearest neighbors (KNN)	0.0%	33.3%
	*K* important neighbors (KIN)	66.7%	100%

Total	Random forest (RF)	38.9%	73.6%
	Support vector machine (SVM)	30.6%	69.4%
	*K* nearest neighbors (KNN)	0.0%	27.8%
	*K* important neighbors (KIN)	32%	72.2%

**Table 3:** Accuracy of different classifiers on benchmark data sets.

Data set	*p*	Train	Test	RF	SVM	KNN	KIN
Connectionist bench	60	104	104	78.7	74.4	71.5	74.0
Ozone	72	102	410	80.6	79.5	72.4	75.4
Prostate cancer	600	70	34	69.1	75.2	66.0	72.3
Colon cancer	2000	32	32	65.7	77.9	62.5	74.3
Liver transplant	39	102	578	88.7	85.7	88.6	89.1
